# A global dataset of inland fisheries expert knowledge

**DOI:** 10.1038/s41597-021-00949-0

**Published:** 2021-07-16

**Authors:** Gretchen L. Stokes, Abigail J. Lynch, Simon Funge-Smith, John Valbo‐Jørgensen, T. Douglas Beard, Benjamin S. Lowe, Jesse P. Wong, Samuel J. Smidt

**Affiliations:** 1 School of Natural Resources and Environment, University of Florida, Gainesville, FL 32611 USA; 2 U.S. Geological Survey, National Climate Adaptation Science Center, Reston, VA 20192 USA; 3 Regional Office for Asia and the Pacific, Food and Agriculture Organization of the United Nations, Bangkok, 10900 Thailand; 4 Fisheries Division, Food and Agriculture Organization of the United Nations, Rome, 00153 Italy; 5 Department of Environmental Science and Policy, George Mason University, Fairfax, VA 22030 USA; 6 Soil and Water Sciences Department, University of Florida, Gainesville, FL 32611 USA

**Keywords:** Environmental impact, Environmental impact, Ichthyology, Freshwater ecology, Sustainability

## Abstract

Inland fisheries and their freshwater habitats face intensifying effects from multiple natural and anthropogenic pressures. Fish harvest and biodiversity data remain largely disparate and severely deficient in many areas, which makes assessing and managing inland fisheries difficult. Expert knowledge is increasingly used to improve and inform biological or vulnerability assessments, especially in data-poor areas. Integrating expert knowledge on the distribution, intensity, and relative influence of human activities can guide natural resource management strategies and institutional resource allocation and prioritization. This paper introduces a dataset summarizing the expert-perceived state of inland fisheries at the basin (fishery) level. An electronic survey distributed to professional networks (June-September 2020) captured expert perceptions (n = 536) of threats, successes, and adaptive capacity to fisheries across 93 hydrological basins, 79 countries, and all major freshwater habitat types. This dataset can be used to address research questions with conservation relevance, including: demographic influences on perceptions of threat, adaptive capacities for climate change, external factors driving multi-stressor interactions, and geospatial threat assessments.

## Abstract (summary)

**Table:** 

Measurement(s)	Perception • Threats • Adaptive capacity
Technology Type(s)	Survey
Factor Type(s)	Fishery location • Demographics
Sample Characteristic - Organism	Homo sapiens • fish
Sample Characteristic - Environment	freshwater environment • lake • stream • wetland ecosystem
Sample Characteristic - Location	Europe • Asia • Oceania • Africa • South America • North America • Australia

Machine-accessible metadata file describing the reported data: 10.6084/m9.figshare.14749161

## Background & Summary

Freshwater fish are important contributors to human livelihoods, food and nutrition, recreation, ecosystem services, and biological diversity. Yet, they inhabit some of the most threatened ecosystems globally^1^, face higher declines relative to marine and terrestrial species^2^, and are disproportionally understudied^3,4^. Inland fisheries are subjected to a suite of anthropogenic stressors across aquatic-terrestrial landscapes^5^, including flow alterations, dams, invasive species, sedimentation, drought, and pollution^6–8^. Evaluating stressors and their impacts on global inland fisheries is essential for effective management, monitoring, and conservation^6^, but unlike marine fisheries, there is no standardized method to assess inland fisheries^9^.

Data inputs for a fisheries threat assessment typically include baseline information, such as species-specific landings or *in situ* population data (volume and composition), size (population and landings), and biomass. In addition, multi-stressor interactions (e.g., synergistic, additive) across complex habitats often warrant cross-ecosystem and cross-sector evaluations at multiple scales^10,11^. However, in the case of inland fisheries, these data inputs are severely deficient and often disparate in many regions^12,13^, which challenges the development of a global assessment. Thus, evaluating stressors and their impacts on inland fisheries necessitates the use of additional data sources (e.g., expert knowledge) beyond those typically derived directly from fish or fish habitats^12,14^. Local and subject-matter expertise can provide contextualized insights where spatial data are limited or unattainable (e.g., emerging threats^15^) and where empirical evidence is incomplete (e.g., multi-stressor interactions).

Expert elicitation (i.e., expert opinion synthesis, where *opinion* is the preliminary state of knowledge of an individual) is increasingly used to inform ecological evaluations and guide water infrastructure, development, food security, and conservation decision-making and assessments, especially in data-poor scenarios^14,16^. While spatial data can be integrated as a suite of individual stressors (i.e., input variables) within ranking systems for the development of vulnerability or habitat assessments for conservation purposes^14,17^, the utilization of spatial variables is limited by the method for determining relative impacts (i.e., value judgment)^18^. Cumulate impact scores and systematic weighted ranking of threats are often based on geographically biased, small sized, or non-representative subsets of experts’ opinions (e.g., global weight determination from eight experts^5^). Thus, data collection for this study was motivated by the development of a global assessment of threats to major inland fisheries, and the overarching need for tractable freshwater indicators. The data generated contribute essential relative influence scores for the assessment and provide a timely snapshot of inland fisheries as perceived by fisheries professionals. Threat composition and influence have broader potential applications to inform vulnerability and adaptation components of freshwater conservation and management targets (e.g., United Nations (UN) Sustainable Development Goals, UN International Decade “Water for Sustainable Development,” Convention on Biological Diversity, Ramsar Convention on Wetlands).

This paper introduces a dataset that can help address a knowledge gap in understanding natural and human influences on inland fisheries with local, contextualized fishery evaluations. Derived from an electronic survey, data comprise perceptions from fisheries professionals (n = 536) on the relative influence and spatial associations of fishery threats, recent successes, and adaptive capacity measures within the respondent’s fishery of expertise.

In the context of the survey, we use the term “threat” as a proximate human activity or process (“direct threat”) causing degradation or impairment (“stress”; e.g., reduced population size, fragmented riparian habitat) to ecological targets (e.g., species, communities, ecosystems; in this case, fishery)^19^. We considered only the threats most proximate and direct to the target (fishery) and excluded stresses (i.e., symptoms, degraded key attributes) and contributing factors (i.e., root causes, underlying factors). For example, we considered pollutants (direct threat) rather than the pollution source (contributing factor) or the resulting contaminated water (stress, effect). We addressed the ambiguity of the term ‘fishery’^20^ by allowing respondents to indicate a geographic location (specific point) within their fishery area. This allows for spatial attribution with an inclusive use of ‘fishery’ as it pertains to threats (e.g., threats to a fish population of fishery-targeted species, catch characteristics, or the habitat in which the fishery operates).

We structured survey questions about the occurrence and relative influence of threats to the production and health of inland fisheries using 29 specified individual threats derived from well-studied pressures to inland fisheries in addition to pressures emerging as threats to fisheries (e.g., climate change, plastics^15^). We categorized individual threats into five well-established categories: habitat degradation, pollution, overexploitation, species invasion, and climate change^1,7^ for organizational context in the survey. We also designed survey questions specifically to understand the social adaptive capacity of fishers using five major community-level domains: fisher *access to assets* (e.g., financial, technological, service), fisher and institutional *flexibility to adapt* to changing conditions (e.g., livelihood alternatives, adaptive management), *social capital and organization* to enable cooperation and collective action (e.g., co-management), *learning and problem-solving* for responding to threats, and fishers’ *sense of agency* to influence and shape actions and outcomes^21^.

This dataset can be useful as an overview assessment, on which future assessments may expand for specific temporal or spatial interests. Some data in this dataset (e.g., microplastics, invasive species disturbances) are otherwise unattainable at relevant scales from geospatial information and therefore provide novel information. Potential uses include demographic influences on threat perceptions, spatial distribution of adaptive capacity measures paired with climate change or other threats, external factors driving multi-stressor interactions, and paired geospatial and expert-derived threat analysis. These data can provide insights on fisheries as a coupled human-natural system and inform regional and global freshwater assessments.

## Methods

### Survey design

**Fig. 1:**
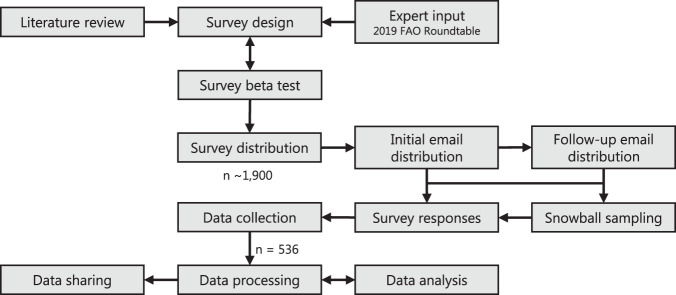
Workflow used to generate and analyse the data outputs, where the survey design (i.e., survey questions, answer choices, and content organization) came from literature review and expert input from the 2019 Food and Agriculture Organization of the United Nations (FAO) Advisory Roundtable on the Assessment of Inland Fisheries^22^; data collection came from responses based on two email distributions and snowball sampling; and data sharing resulted from data processing and analyses.

The questions and response choices in this survey were deliberately selected, designed, and tested using literature review and expert input (Fig. 1). We derived individual threats (i.e., proximate pressures, direct threats) from established, documented threats to inland fishery populations and habitats^7^ plus additional documented emerging threats (e.g., climate change, microplastic pollution^15^). We cross-referenced answer choices using peer-reviewed literature to ensure consistent word choice and adequate justification. We solicited additional input from fisheries experts on threat scoring mechanisms and fishery syntax during the Food and Agriculture Organization of the United Nations (FAO) Second Advisory Roundtable on the Assessment of Inland Fisheries^22^. We used Roundtable feedback to create the survey. Prior to distribution, we made a few additional modifications to the survey design using beta test (i.e., pre-test) feedback from fisheries professionals not involved in the survey.

The survey used to generate this dataset comprised five sections pertaining to the respondent’s fishery of expertise: 1) geographic location, 2) threats, 3) successes, 4) adaptive capacity, and 5) demographics (Table 1, Appendix A). Respondents could provide optional, additional comments. Respondents indicated the location of their self-identified fishery (basin) of expertise by one or both of the following: a) clicking a point (pin drop) inside their fishery’s water body using a *Google Maps* extension (recorded as geographic coordinates) and/or b) selecting their region, and/or subregion name from a list of provided choices. The *threats* section had three components: a) overall perceived threat of the respondent’s fishery, as indicated by moving a gauge (0 to 10; 0 = not threatened to 10 = highly threatened), b) types of threats present in the fishery (where respondents checked all threats that apply to their fishery from a given list with an option to add additional threats), and c) relative influence of each threat selected in the previous question to the total threat (must add up to 100%). Part “b” of the *threats* section also included a practice question prior to the threat gauge question, intended to help respondents learn how to use the gauge (see *Technical Validation*). Respondents were asked to describe one recent success in their fishery for the *successes* section, which were recorded as open-ended text responses. The *adaptive capacity* section used a Likert scale (strongly disagree, somewhat disagree, neither disagree nor agree, somewhat agree, strongly agree) for five domains of adaptive capacity: access to assets, flexibility to adapt, social capital/organization, learning and problem solving, and sense of agency. Finally, the *demographics* section included the following components: current affiliation, primary area of expertise, years of fisheries experience, proportion of work time spent in a field-based setting, highest degree earned, birth year, and sex (each selected from a list of provided options). At the conclusion of the survey, respondents were thanked and given the survey link in the case they wanted to take the survey again for a different basin of expertise; respondents were allowed to take the survey more than once.

**Table 1:** Survey structure, categories and content of questions asked. All data collection was performed using *Qualtrics* (Appendix A).

Survey section	Question components
Fishery location (3 questions)	Region
	Subregion
	Geographic coordinates (inside fishery area)
Threats (4 questions)	Overall threat to fishery
	Types of threats present in fishery
	Relative influence of individual threats
Adaptive capacity (5 questions)	Assets
	Adaptability
	Flexibility
	Cooperation
	Agency
Successes (1 question)	Recent fishery success
Demographics (7 questions)	Affiliation
	Area of expertise
	Proportion of time in a field-based setting
	Years of fisheries experience
	Sex
	Birth year
	Education level

### Survey distribution & data collection

We distributed a *Qualtrics* survey via anonymous link to ~1,900 inland fisheries professionals, including: ~1,250 American Fisheries Society members (Fish Habitat, Canadian Aquatic Resources, and International Fisheries Sections), ~500 FAO affiliates and collaborators, and ~150 InFish network members^23^. We sent an initial email (including survey link, instructions, project summary) on June 16, 2020 and a follow-up reminder email (above materials plus QR code and survey flier) on July 8, 2020. The initial distribution intentionally targeted three fisheries organizations where membership or affiliation reflects some level of fisheries experience or leadership (i.e., members can be considered fisheries professionals by way of affiliation or membership criteria). Distribution was not limited to fisheries professionals from any one type of inland fishery or fisheries sector. Snowball sampling was permitted to increase representation of fisheries professionals not affiliated with the targeted organizations. Survey respondents were encouraged to share the survey with their colleagues in corresponding organizations. Surveys were available in English, Spanish, Portuguese, French, Korean, and Chinese. Survey respondents could select their language of preference upon opening the survey. Data collection occurred June 16 - September 9, 2020, with 98% (n = 524) of the 536 total responses in the dataset (i.e., responses that met the criteria for inclusion (see *Data Validation*)) occurring in the first month (June 16 - July 15, 2020).

### Institutional board review and informed consent

This study (#IRB202000533) was approved as ‘Exempt’ by the University of Florida Gainesville Campus (IRB-02) Institutional Review Board (UF IRB) on May 15, 2020 (Appendix B). As no identifying information was collected, UF IRB approved a waiver of documentation of informed consent for this study. Respondents were instead presented with a written informed consent statement immediately before beginning the survey, and their consent was implied by their participation in the survey. In accordance with IRB policies, all respondents were at least 18 years old. The purpose of the study, estimated time for completion, additional instructions for respondents who wanted to complete the survey for more than one area of expertise, potential risks, and contact information of the principal investigator (S. Smidt), co-investigator (G. Stokes), and UF IRB were provided to respondents before asking about their consent. The introductory text highlighted no potential risks to respondents: “There are no risks anticipated in participating in this survey nor any direct benefits or compensation. However, the results will be a valuable contribution to improving global inland fisheries assessment with local applications. All responses will remain completely anonymous and no identifying information will be collected. You may stop this survey at any time and you can decline to answer questions as you wish.” See Appendix A for additional information provided to respondents.

Survey participation did not explicitly exclude anyone; however, respondents were likely to be self-selecting, where those who felt comfortable answering the questions and considered themselves eligible after reading the study description were more likely to take the survey. Emails sent to prospective respondents included the following information: rationale/study overview, purpose, importance, IRB approval number, and what to expect if they chose to take the survey (e.g., time estimate).

### Data processing

**Fig. 2:**
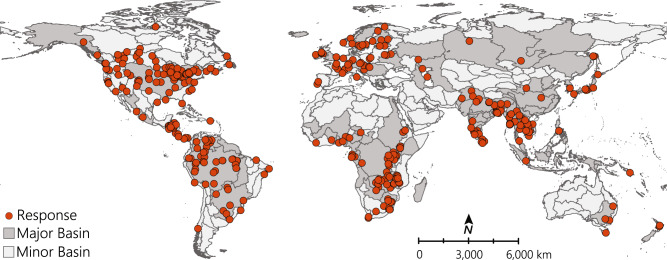
The global distribution of georeferenced responses (orange circles; n = 432) in the dataset, excluding those without geographic coordinates (n = 104; region or subregion only), where major basins (dark grey) represent hydrological basins accounting for 95% of inland fish catch;^32^ and minor basins (light grey) represent all other hydrological basins (HydroBASINS level 3^33^).

**Fig. 3:**
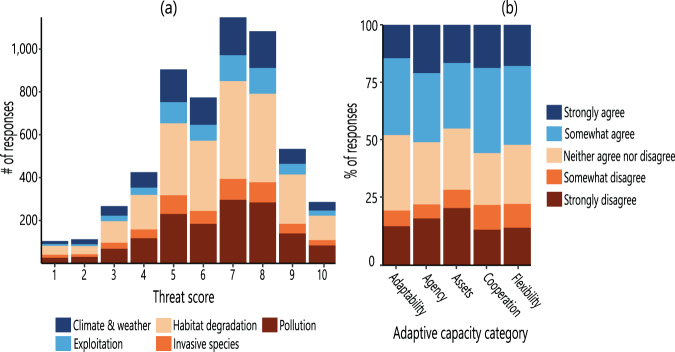
Survey responses summarized by (**a**) threat score counts by threat category and (**b**) adaptive capacity domains by response type. (**a**) shows the total response counts of the number of provided threats (n = 29) selected in respondents’ fisheries (range = 0–29), summed by major threat category (n = 5; fill colors) across overall fishery threat scores (1–10; x-axis). (**b**) depicts adaptive capacity domains in respondents’ fisheries (n = 467, except “assets” = 466), shown as the percent of responses in each adaptive capacity domain, and colored according to respondent answers (Likert scale; five categorical choices from strongly disagree to strongly agree).

We received 712 responses between June 16 and September 9, 2020. All provided language options were utilized by respondents: English (n = 565), Spanish (n = 81), French (n = 26), Korean (n = 2), Portuguese (n = 35), Chinese (n = 3; traditional = 2, simplified = 1). We exported data from *Qualtrics* using numeric values, with line breaks removed and multi-value fields split into columns. Data were imported using the package *qualtRics* in R (R Development Core Team 2018)^24^. We used the following dataset inclusion criteria for responses in the raw dataset: 1) submission date after the official start of the survey (“enddate” > 06/16/2020 00:00), 2) fishery location provided (one or more of the following: region, subregion, geographic coordinates), 3) at least 15% of the survey completed (“progress” ≥ 15), and 4) a response to the overall threat question (“overall_threat” ≠ “NA”) (see *Data Validation*). The latter was an indicator of respondent completion, assuming that if the first question of the survey after location was incomplete, then the rest of the survey was not likely adequately considered. Responses flagged as potential spam or recorded as a survey preview were already removed with the above criteria. Responses that did not meet the inclusion criteria (n = 176) are not included in the dataset. We processed data into a formatted dataset file to enhance its interpretability and usability with the following steps: 1) renamed column names with descriptive properties, 2) recoded numeric values to character choice options where applicable, 3) extracted spatial coordinates (longitude, latitude) of fishery locations, and 4) added translations for non-English entries. We back-translated survey responses (i.e., “other” columns for affiliation and area of expertise) that were recorded in languages other than English using the *Qualtrics* Google Translate function. Additional processing for figures included geospatial visualization of responses (Fig. 2), and summation and averaging of threat data to generate threat counts by threat categories and total threat counts (Fig. 3a,b). All R code used to generate the formatted data and figures is available for users in HydroShare^25^.

## Data Records

The inland fisheries expert knowledge dataset is provided as a CSV and Excel (.xlsx) file in two forms: raw and formatted. The raw dataset contains data as recorded by *Qualtrics*, intended as an original reference dataset. The formatted dataset, intended for reuse, contains the same data with organizational modifications (see *Methods)*. Each row in the data file represents one completed survey by an individual respondent and each column represents a question or component thereof. A full description of each column and its units or format is provided as an additional CSV and Excel (.xlsx) file, which is intended to extend the reuse potential of the dataset with full variable explanations. While the survey did not solicit identifying information, some optional text entries provided by respondents for “successes” and “comments” contain information that could potentially be used to trace to an individual respondent and thus are excluded from the main dataset in accordance with UF IRB protocol requirements to protect respondent confidentiality. An independent, compliant file of text entries disassociated from geographic information and with proper names removed is available upon request. The raw and formatted dataset and accompanying reference data are freely available to the public and can be accessed in the Consortium of Universities for the Advancement of Hydrologic Science, Inc. (CUAHSI) HydroShare data repository^25^. HydroShare access requires users to sign up for a free user account (https://www.hydroshare.org/sign-up/) with organization name, user type, email, username, password, country, and state; once registered, users may log in for data access. Figures depict the spatial representation of responses (Fig. 2), response distribution of relative threat influences by threat category (Fig. 3a), and adaptive capacity measures by domain type (Fig. 3b). Tables provide data summaries of responses: survey structure and content (Table 1), response distribution by region and language (Table 2), relative influence of threats by category (Table 3), summary of individual threat influences and counts by category (Table 4), and respondent biographical information (Table 5).

**Table 2:** Survey response distribution (counts, proportion) by region and language (n = 536).

Region	Language					Count	Proportion
	English	Spanish	French	Portuguese	Chinese		
North America	120	0	0	0	0	120	0.23
Asia	114	0	0	0	1	115	0.22
Africa	106	0	7	2	0	115	0.22
South America	8	34	0	28	0	70	0.13
Europe	53	0	13	1	0	67	0.13
Central America & Caribbean	3	27	0	0	0	30	0.06
Oceania	10	0	0	0	0	10	0.02
Central Asia	1	0	0	0	0	1	0.00
Middle East	1	1	0	0	0	2	0.00
*Unspecified*	3	3	0	0	0	6	0.01
**Total count**	419	65	20	31	1	536	1.00

**Table 3:** Aggregated mean and relative influence (excluding “other” threats) of threat categories by percent of total threats (see Table 4).

Category	Mean	Relative Influence
Habitat Loss	11.78	0.22
Pollution	8.51	0.16
Invasive Species	9.92	0.18
Exploitation	15.58	0.29
Weather and Climate	8.28	0.15

**Table 4:** Individual threats by percent of responses, where *mean* (%) is the averaged threat percent for each category out of the total threat, with standard deviation, *SD*; *max* (%) is the maximum percent contribution of each individual threat to total threat (0–100%); and *count* is the number of respondents who selected each individual threat as a threat to their fishery.

Threat category	Individual threat	Mean	SD	Max	Count
**Habitat loss** *Degradation*	Deforestation and associated sediment runoff	13.02	13.22	100	304
	Riparian loss, degradation	10.30	9.75	60	301
	Channelization	7.41	8.05	59	152
	Dredging	4.99	5.77	28	89
**Habitat loss** *Hydrological alterations*	Wetland drainage	8.07	8.56	75	215
	Dams	16.01	15.08	83	291
	Weirs	8.57	9.00	50	122
	Other flood protection	5.15	5.83	40	92
	Extraction for agriculture	8.07	9.56	94	220
	Extraction for industry	4.20	5.17	31	116
	Extraction for urban use	4.59	4.36	21	146
**Pollution**	Agricultural effluents	8.55	9.26	82	339
	Industrial effluents	7.00	7.28	40	186
	Urban wastewater	6.44	6.36	34	269
	Aquaculture effluents	5.22	5.88	27	88
	Plastics	4.47	4.36	28	167
	Pharmaceuticals	2.57	3.05	20	72
	Oil or gas exploration	7.27	7.67	35	71
	Mining	9.49	10.10	62	162
**Invasive species**	Invasive non-native species	10.73	13.20	91	333
	Problematic native species	4.84	6.81	40	73
	Introduced genetic material	3.53	4.23	25	58
**Exploitation**	Overfishing	17.93	16.96	100	287
	Destructive fishing practices	12.82	13.36	95	245
**Weather and climate**	Change in water temperature	7.31	8.25	60	233
	Change in wind patterns	3.65	4.78	23	48
	Change in flooding (extent, timing)	7.68	7.53	40	270
	Drought	9.13	10.65	100	216
	Change in ice cover	6.06	6.52	35	62
**Other**	Other	15.71	16.08	100	63

**Table 5:** Respondent biographical information by response counts and response proportion per category.

Affiliation (n = 464)	Count	Proportion	Fisheries experience (n = 464)	Count	Proportion
Government	126	0.27	<5 years	48	0.10
University	152	0.33	5–10 years	72	0.16
Non-governmental organization	92	0.20	10–15 years	83	0.18
For-profit enterprise	15	0.03	15–20 years	56	0.12
Fisher association	9	0.02	>20 years	205	0.44
Tribal affiliate	2	0.00	**Sex (n** = **461)**		
Retired	29	0.06	Male	355	0.77
Other	39	0.08	Female	106	0.23
**Education level (n** = **465)**			**Field time (n** = **465)**		
Some college courses	2	0.00	None	21	0.05
Associate’s degree	2	0.00	A little time (<10%)	103	0.22
Bachelor’s degree	60	0.13	Some time (10–50%)	185	0.40
Master’s degree	157	0.34	A lot of time (50–80%)	99	0.21
Doctoral degree	244	0.52	Most of the time (>80%)	57	0.12
**Area of expertise (n** = **466)**			**Age (n** = 457)*		
Fishery management	160	0.34	<25	7	0.02
Research - genetics**	21	0.04	25–34	73	0.16
Research - ecology	144	0.31	35–44	99	0.22
Environmental monitoring	29	0.06	45–54	113	0.25
Aquaculture	31	0.07	55–64	111	0.25
Extension/outreach	10	0.02	65–74	49	0.11
Policy	17	0.04	75+	5	0.01
Fishing	23	0.05			
Other	31	0.07			

*mean = 48.4 ± 13.1.

**includes evolutionary biology.

## Technical Validation

We evaluated sources of error within the Total Survey Error framework^26,27^ by examining potential errors in the: 1) survey instrument and design (errors of observation), 2) sampling and coverage (errors of non-observation), and 3) data processing (errors of processing).

### Survey instrument and design

We used the *Qualtrics* “quality control” tool (ExpertReview by iQ) to assess the survey for usability and accessibility. Most Web Content Accessibility Guidelines (WCAG) issues were scored as “minor,” indicating minimal error introduced across various modes of survey access (e.g., computer, smartphone). No issues in the “survey error” metrics were identified. “Methodology” metrics were scored as “minor” for the use of more than one text entry box (necessary for “other” choice entry).

To the extent possible, we used wording for survey questions from well-established international or national protocols and assessments. Questions were based directly on peer-reviewed literature, and when possible in the English version, used exact wording as provided in the literature to minimize bias in survey results; wording may not align as precisely in translated versions (despite the four-step, two-way translation process, see below). Threat categories and names were derived from well-established sources^7,17^ with minor additions based on evidence of emerging threats^15^, and adaptive capacity domains were derived from Cinner *et al*.^21^.

A possible source of error is the respondent’s interpretation of threat and their ability to correctly use the gauge tool to indicate the level of threat in their fishery (see Appendix A, Question 3). Respondents were asked to move a needle on a gauge tool until the number on the dial matched the number of their perceived level of threat (i.e., gauge needle pointed far left = no threat (0), gauge needle pointed far right 180 degrees = high threat (10)). The functionality of the tool may have introduced a potential barrier for respondents in correctly indicating or interpreting the threat level of their fishery. We aimed to reduce these effects by including a practice question prior to the threat question, which asked the same question, but using a hypothetical fishery example. Respondents were instructed to move a gauge according to how they perceived threats in a hypothetical fishery; their answer to this question indicates the respondent’s understanding of directionality of the gauge. We expected respondents to score the hypothetical fishery on the left side of the gauge (0 to 5); over 75% of respondents did so. We recommend data users apply caution when utilizing threat scores for the remaining responses with hypothetical fishery scores greater than 5. Users may also choose to create a weight or a reliability score for the threat score based on the congruence between the response to the test question and the threat score.

Additionally, “threat” was intentionally used broadly (undefined in the survey itself) to allow for respondent consideration of a suite of pressures on their individual fishery. However, we recognize that what one person may consider “highly threatened” (e.g., threat score of 10) another person may consider only “moderately threatened” (e.g., threat score of 5). Similarly, the use of Likert-scale responses for adaptive capacity measures may introduce some variability, as one person’s “strongly agree” may be different from another person’s interpretation of the same phrase^28^. We expect these issues to dissipate (i.e., average out) across the large sample size and across same-scale measures (e.g., Cronbach’s Alpha = 0.839 for the five Likert-scale items). We recommend that data users apply the appropriate statistical tests for reliability and variability that fit their desired analyses and, if deemed appropriate, some users may wish to aggregate Likert-scale or threat rating scale responses into binary scales. We mitigated potential error in survey question translations using a four-step, two-way translation process: 1) preliminary translation to each designated language using Google Translate; 2) translations revised or rewritten by two independent, native (fluent) language speakers; 3) independent translations back-translated to English and compared for discretions; and, if further review was warranted, 4) review of final translations in survey formatting by a native speaker.

*Qualtrics* is generally accessible worldwide, but internet censorship in some countries may restrict access and thus, survey participation. To reduce access issues, UF IRB approved a method to obtain and record responses, while maintaining anonymity, from anyone who was unable to access the survey link directly. The respondent who elected this option (n = 1) typed answers on a PDF version of the survey and returned it to the approved survey team member via email. The team member removed all identifying information from the form and sent the anonymous version of the completed survey to a different approved team member, who entered the information into *Qualtrics* via anonymous survey link.

### Sampling and coverage

The purpose of this study as a global snapshot of inland fisheries and the lack of a source location for all inland fisheries professionals warranted the use of snowball sampling to recruit professionals outside major membership or affiliation networks (i.e., hidden populations). However, snowball sampling inherently introduces sampling bias to the dataset. The initial target population has a strong impact on the representativeness of the snowballed sample. Sourcing the initial target population from three well-established inland fisheries networks (FAO, InFish, American Fisheries Society) with diverse spatial and demographic representation was the best defence against this potential bias^29,30^. Initial respondents (i.e., those receiving a direct email from the study team with the survey link) were encouraged to share the survey within their networks or to specific contacts, which they believed met the survey criteria. We improved spatial coverage of snowballing by indicating basins lacking responses in areas where fisheries professionals would be expected (i.e., in the major basins that capture 95% of global inland fish catch^31^) in a follow-up email sent to initial respondents, with encouragement for snowball sampling in those areas. Responses included in this dataset comprise basins that capture 82.1% of reported global inland fish catch^32^. The initial respondents may share certain characteristics or affiliations with snowballed respondents, which could skew the total representation of respondents to be unrepresentative of the larger population of inland fisheries professionals. As such, contextualizing responses within this potentially skewed population sample is important for analysis. Responses should not be extrapolated as representative of an entire basin or region, or as an indicator of historical or future threats, but rather as a unique, discrete geographic snapshot.

Initial survey distribution intentionally targeted respondents whose membership or affiliation reflects some fisheries experience. Those who read the description of the target respondents for the survey and felt they did not fit the criteria were likely self-excluded from the sample population. However, we recommend that data users subsample the dataset using the demographic parameters fit for their study needs. For example, a user could define ‘expert’ using specific criteria (e.g., >15 years of experience in fisheries with a doctoral-level degree) and subsample accordingly.

A limitation of this dataset is that the global scope limits localized resolution. Given the global scope, we do not assess characteristics of the respondent’s fishery (i.e., no information collected about the types of fishes or type of fishery considered by the respondent while taking the survey). Although we do not specify temporal or spatial constraints for respondents to consider, consistent use of present tense in the survey implies current threat (as was the case in pre-testing). Follow-up studies may use the information from this dataset to expand upon temporal characteristics of pertinent threats, such as the onset, duration, severity, or spatial expanse. Potential biases from respondent interpretations of the questions over variable time scales and size of fishery areas may limit extrapolation of the data to geospatially derived attributes beyond the participant-selected geographic coordinates. The specificity of a single geographic point associated with each entry in the dataset, however, allows for precise attribution to that point, to which any extrapolation or generalization can be traced.

### Data processing

Specified inclusion criteria were applied for the dataset creation (see *Methods* section). Survey responses marked as survey preview or spam were discarded from the dataset (n = 6). Survey preview responses were incomplete test responses recorded by the study team. *Qualtrics* flags responses as potential ‘spam’ if two or more identical responses are received from the same IP address in a 12-hour period. We ensured that threat influence responses totalled to 100% using the “Custom Validation” function in *Qualtrics*, where we specified that the percent of total threat caused by each individual threat must add up to 100, and enabled grid lines and percent labels to improve accuracy of percent choices by respondents (Appendix A). Respondents were permitted to change the slider bars for all individual threats until they were satisfied with the percentage allotted to each and they totalled to 100. We confirmed this summation in the dataset by adding the columns associated with the relative influence of each individual threat (n = 30); we found no errors. Additionally, we performed quality checks of the percent influence of individual threats by aggregating responses by region and individual threats. Based on a less than 8% variation in standard deviation across regions and similar minimum and maximum values across all threat types, we do not see evidence of bias in the way the slider tool was used for different regions or for different threat types. Aside from accidental response selection by respondents, the electronic format of the survey and automated processing software restricted errors from coding or human entry error.

## Usage Notes

This dataset can be used to answer a diverse array of scientific questions and provide valuable insights about the social-ecological dynamics of inland fisheries at a global scale. It may also enhance existing ecological or spatial datasets of similar metrics. Questions of interest may include:

1. What types of anthropogenic stressors pose the greatest direct threat to inland fisheries, as perceived by experts?
2. How are threat types, and the relative influence of each, distributed across space (e.g., basin, continent, habitat type)? Do threats trend together based on fishery characteristics of geography?
3. How do threats relate to fish catch, dependence on fisheries for food/income, and/or human development (e.g., Human Development Index, Multidimensional Poverty Index)?
4. How do perceived threats relate to remotely sensed data records of similar types (e.g., deforestation, dams, mining)? For example, do areas where dams were scored as having the highest relative influence relate to density of dams, dam size, or reservoir capacity?
5. Are fisheries perceived to be most threatened equipped with greater adaptive capacity measures (i.e., five adaptive capacity domains) (e.g., Cinner *et al*.)^21^?
6. How is adaptive capacity linked to socioeconomic parameters (e.g., gross domestic product, governance)?
7. What key demographic attributes characterize areas of higher or lower threat (i.e., how do threat scores differ across demographic categories: affiliation, expertise, years of experience working in fisheries, field time, age, sex, and education level)?
8. How are threats related to each other (i.e., are any threats more likely to be present with the occurrence of other threats)?
9. What is the influence of threats for which no other global datasets exist (e.g., microplastics) and how might the data from this dataset fill data gaps?
10. Do the fisheries with highest perceived climate threats align with regions of documented climate change or projected substantial change?

This dataset contains geospatial information on the location of the fishery associated with each survey response. 81% of responses (n = 432) are associated with geographic coordinates (latitude and longitude) of a point inside the respondent’s fishery, 19% of responses (n = 102) are associated with both a region (basin) and subregion (sub-basin), and 2 responses have a region only (no subregion). As such, all responses can be linked to hydrological basins (HydroBASINS level 3)^33^ and most responses can be spatially linked to data in other datasets by coordinate points. To obtain additional information about the location, such as water body type, country, or ecoregion, the user must project the data using the geographic reference system WGS 1984, then join attributes from features in additional datasets to this dataset based on their spatial relationship. The user may set join specifications (e.g., boundaries touching, closest to point) to best suit their questions of interest. To join non-georeferenced survey responses to hydrological basins, users may join basin and sub-basin names to HydroBASINS levels 3 and 4, respectively.

## Supplementary information

Appendix B

Appendix A

## Data Availability

Code used to format the dataset for reuse and generate manuscript figures (Fig. 3a,b) is available for download in the Consortium of Universities for the Advancement of Hydrologic Science, Inc. (CUAHSI) HydroShare data repository at 10.4211/hs.de4190f0eff74b09a5e0844a0de482a5^25^. Note that, for Fig. 2, this also requires downloading hydrological basin from HydroBASINS^33^ and catch data from FAO^32^. Upon free registration in HydroShare, there are no restrictions to the access or use of this code. Code was implemented in R (version 4.0.2; https://r-project.org)^24^.
