# Supplementary material for: A global dataset of inland fisheries expert knowledge
Source: Sci Data. 2021 Jul 16;8:182. doi: 10.1038/s41597-021-00949-0 (PMC8285391; doi:10.1038/s41597-021-00949-0)
Supplement: Supplementary file 1 — Appendix B [file 41597_2021_949_MOESM1_ESM.pdf]

Appendix B. Study approval letter from the University of Florida Institutional Review Board

Institutional Review Board
UNIVERSITY of FLORIDA

Behavioral/NonMedical Institutional Review Board
FWA00005790

PO Box 112250
Gainesville FL 32611-2250
Telephone: (352) 392–0433
Facsimile: (352) 392–9234
Email: irb@ufl.edu

DATE: 5/15/2020
TO: Samuel Smidt
2181 McCarty Hall A
Gainesville , Florida 32611
FROM: Ira Fischler, Ph.D., Professor Emeritus
Chair IRB-02
IRB#: **IRB202000533**
TITLE: Assessing Expert opinion on basin-level threats to inland fisheries

Approved as Exempt

You have received IRB approval to conduct the above-listed research project. Approval of this project was granted on 5/15/2020 by IRB-02. This study is approved as exempt because it poses minimal risk and is approved under the following exempt category/categories:

> 2. Research that includes only interactions involving educational tests (cognitive, diagnostic, aptitude, achievement), survey procedures, interview procedures, or observation of public behavior (including visual or auditory recording) if at least one of 3 criteria are met: (i) the information obtained is recorded by the investigator in such a manner that the identity of the human subjects cannot readily be ascertained, directly or through identifiers linked to the subjects; (ii) any disclosure of the human subjects' responses outside the research would not reasonably place the subjects at risk of criminal or civil liability or be damaging to the subjects' financial standing, employability, educational advancement, or reputation; OR (iii) the information obtained is recorded by the investigator in such a manner that the identity of human subjects can readily be ascertained, directly or through identifiers linked to the subjects, and an IRB conducts a limited review to make the determination required by 45 CFR 46.111(a)(7) (which relate to there being adequate provisions for protecting privacy and maintaining confidentiality) AND the research is not subject to subpart D.

Special Note(s) to Investigator:

In the myIRB system, exempt approved studies will not have an approval stamp on the

consents, fliers, emails, etc. However, the documents reviewed are the ones to be used. If you need to modify the document(s) in any manner then you'd need to submit to our office for review and approval prior to implementation.

> Please review this Institutional Guideline to determine if you can in fact continue your activities and/or enroll participants:
>
> https://research.ufl.edu/wp-content/uploads/humanresearchcovid.pdf

Principal Investigator Responsibilities:

The PI is responsible for the conduct of the study.

- Using currently approved consent form to enroll subjects (if applicable)
- Obtaining approval for revisions before implementation
- Reporting Adverse Events
- Retention of Research Records
- Obtaining approval to conduct research at the VA
- Notifying other parties about this project's approval status

Should the nature of the study change or you need to revise the protocol in any manner please contact this office prior to implementation at 352-392-0433 or via email, irb@ufl.edu

Study Team:

Benjamin	Lowe	Co-Investigator
Gretchen	Stokes	Co-Investigator

The Foundation for The Gator Nation
An Equal Opportunity Institution
Confidentiality Notice: This e-mail message, including any attachments, is for the sole use of the intended recipient(s), and may contain legally privileged or confidential information. Any other distribution, copying, or disclosure is strictly prohibited. If you are not the intended recipient, please notify the sender and destroy this message immediately. Unauthorized access to confidential information is subject to federal and state laws and could result in personal liability, fines, and imprisonment. Thank you.
