# Supplementary material for: A global dataset of inland fisheries expert knowledge
Source: Sci Data. 2021 Jul 16;8:182. doi: 10.1038/s41597-021-00949-0 (PMC8285391; doi:10.1038/s41597-021-00949-0)
Supplement: Supplementary file 2 — Appendix A [file 41597_2021_949_MOESM2_ESM.pdf]

Appendix A. Survey questionnaire, as it appeared to participants in *Qualtrics.*

UF | UNIVERSITY of FLORIDA

English

Thank you for agreeing to take part in this short survey (**~5 minutes**) about inland fisheries. Your feedback is **integral** to the development of a global assessment framework. Your expertise will help us understand how to **score relative threats** of environmental and fishing pressures and **identify bright spots** of recent adaptability and successes.

If you work in **more than one basin** or fishery, you may take this survey multiple times for each fishery/area where you work. At the end of this survey, you will be given the opportunity to take it again if you wish.

There are **no risks** anticipated in participating in this survey nor any direct benefits or compensation. However, the results will be a **valuable contribution** to improving global inland fisheries assessment with local applications. All responses will remain **completely anonymous** and no identifying information will be collected. You may stop this survey at any time and you can decline to answer questions as you wish. For information regarding your rights as a research participant, contact the University of Florida Institutional Review Board at irb2@ufl.edu. For questions or more information, please contact Sam Smidt (ssmidt@ufl.edu) or Gretchen Stokes (gstokes@ufl.edu).

By clicking "NEXT," you confirm you are at least 18 years old and give your consent to participate in this survey.

Next

English

Where is your **region** of fisheries expertise? Please select "Other" if not listed, and describe below.

Region

Sub-Region / Basin

Please **specify the location** of your fishery by typing it in the box below or moving the pin within the water body. *You can zoom in and out of the map to navigate to your fishery.*

Enter a location

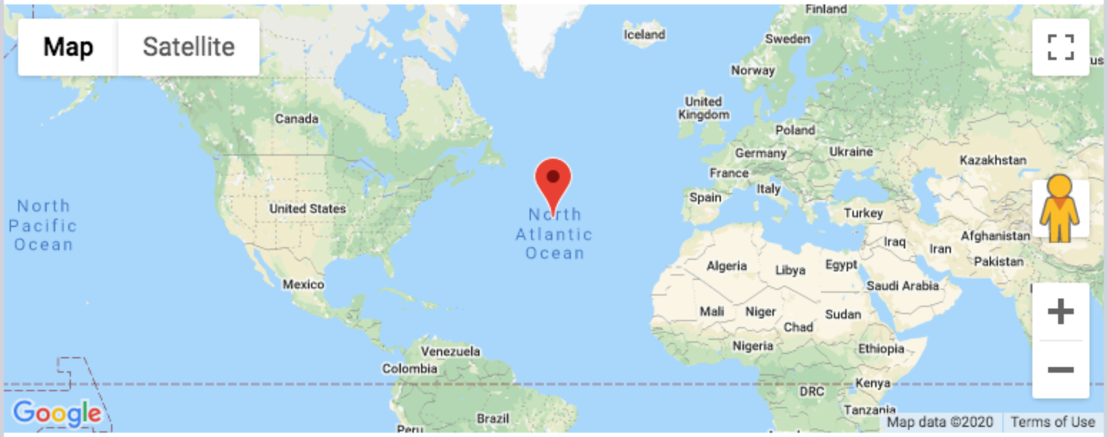

←

Next

English

Imagine a **hypothetical fishery** that has a healthy stock status within well-protected habitat. Based on the information provided, what score would you give for the **level of threat** of this hypothetical fishery, where **1** is **least threatened** (very good condition) and **10** is **most threatened** (very bad condition)?

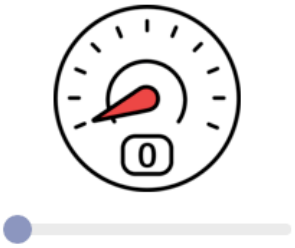

What is the **level of threat** to fisheries in **your area**, where **1** is **least threatened** (very good condition) and **10** is **most threatened** (very bad condition)?

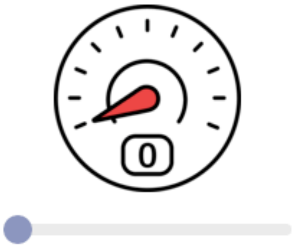

←

Next

Which of the following are direct **threats*** to fisheries in your area? *Please check all that apply.*

**Derived from Arthington et al. 2016 & Salafsky 2008*

**Habitat Loss** Degradation	Deforestation & associated sediment runoff	Riparian loss, degradation	Channelization	Dredging
**Habitat Loss** Hydrological alterations	Wetland drainage	Dams	Weirs	Other flood protection
	Extraction for agriculture	Extraction for industry	Extraction for urban use	
**Pollution**	Agricultural effluents	Industrial effluents	Urban waste water	Aquaculture effluents
	Plastics	Pharmaceuticals	Oil or gas exploration	Mining
**Invasive Species**	Invasive non-native species	Problematic native species	Introduced genetic material	
**Exploitation**	Overfishing	Destructive fishing practices		
**Weather & Climate**	Change in water temperature	Change in wind patterns	Change in flooding (timing, extent)	Drought
	Change in ice cover			

Other (please list):

←

Next

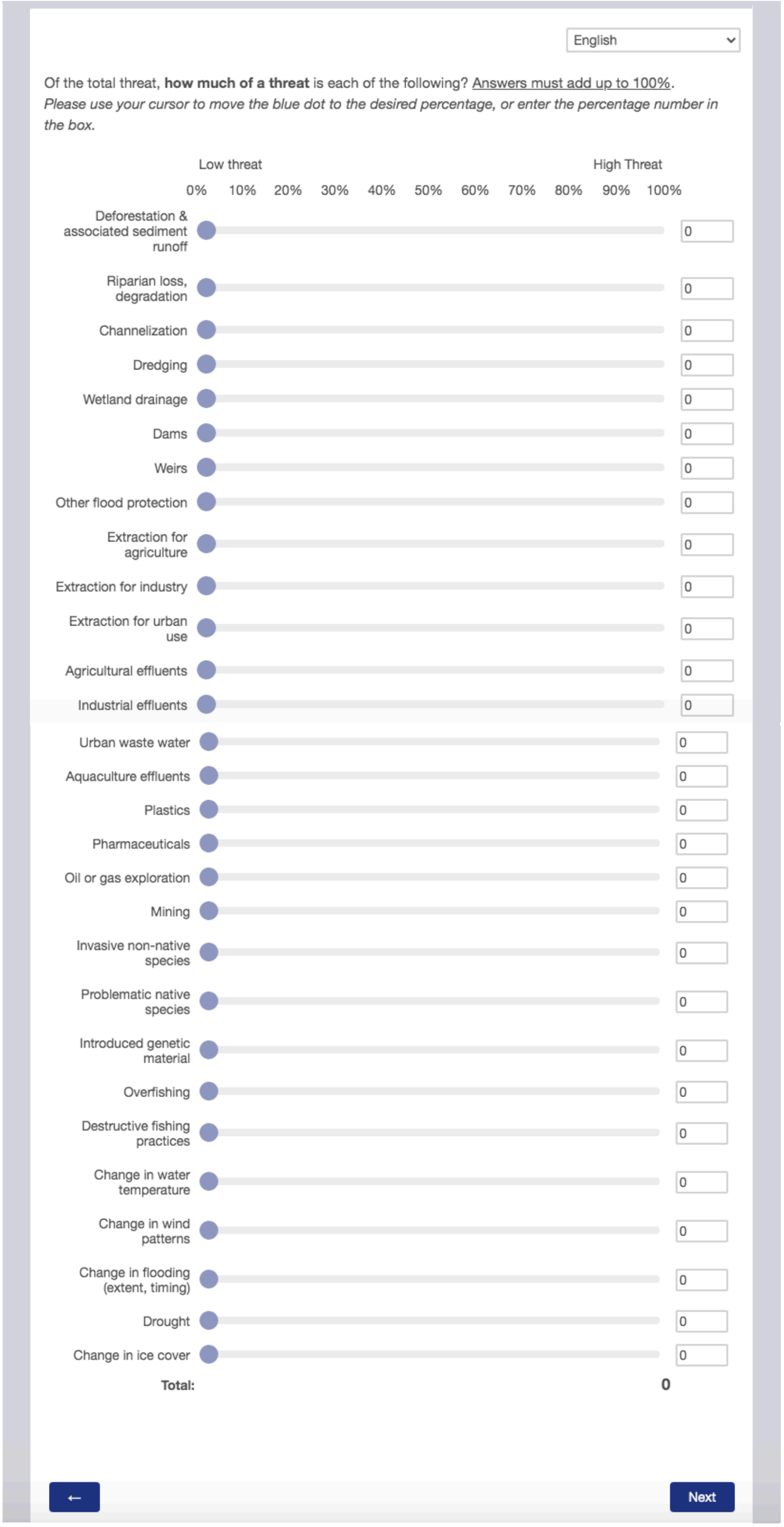
English
Of the total threat, **how much of a threat** is each of the following? Answers must add up to 100%.
Please use your cursor to move the blue dot to the desired percentage, or enter the percentage number in the box.
Low threat
High Threat
0% 10% 20% 30% 40% 50% 60% 70% 80% 90% 100%
Deforestation & associated sediment runoff
Riparian loss, degradation
Channelization
Dredging
Wetland drainage
Dams
Weirs
Other flood protection
Extraction for agriculture
Extraction for industry
Extraction for urban use
Agricultural effluents
Industrial effluents
Urban waste water
Aquaculture effluents
Plastics
Pharmaceuticals
Oil or gas exploration
Mining
Invasive non-native species
Problematic native species
Introduced genetic material
Overfishing
Destructive fishing practices
Change in water temperature
Change in wind patterns
Change in flooding (extent, timing)
Drought
Change in ice cover
Total:
0
←
Next

How much do you **agree** or **disagree** with the following statements about **your fishery**? *Please choose the best option.*

	Strongly disagree	Somewhat disagree	Neither agree nor disagree	Somewhat agree	Strongly agree
Fishers have **access to assets** (e.g. financial, technological, service)	O	O	O	O	O
Fishers and institutions have **flexibility to adapt** to changing conditions (e.g. livelihood alternatives, adaptive management)	O	O	O	O	O
My fishery has **effective social capital / organization** that enables cooperation and collective action (e.g. co-management)	O	O	O	O	O
**Learning** and **problem solving** are encouraged for responding to threats and changes in the fishery	O	O	O	O	O
Fishers have a **sense of agency** (i.e. freedom, power) to influence and shape actions and outcomes	O	O	O	O	O

Lastly, please briefly describe a **promising success** from your fishery in the last 5 years.
For example, this could include successes in stock assessment, biodiversity conservation, habitat conservation, management, governance or social dimensions.

What is your **current affiliation**?

- Government
- University
- Non-governmental organization
- For-profit enterprise
- Fisher association
- Tribal affiliate
- Retired
- Other

What is your primary **area of expertise**?

- Fishery management
- Extension / Outreach
- Environmental monitoring
- Aquaculture
- Research - genetics, taxonomy, evolutionary biology
- Research - ecology
- Policy
- Fishing
- Other

What proportion of work time do you spend **working directly with fish or fishers** in a field-based setting?

- None
- A little time (<10%)
- Some time (10-50%)
- A lot of time (50-80%)
- Most of the time (>80%)

How many **years of experience** do you have working in fisheries?

- <5
- 5-10
- 10-15
- 15-20
- >20

What is your **sex**?

- Male
- Female

What is your **birth year**?

What is your **highest degree of education**?

- High school diploma
- Some college courses
- Associate's degree
- Bachelor's degree
- Master's degree
- Doctoral degree

←

Go to Final Page

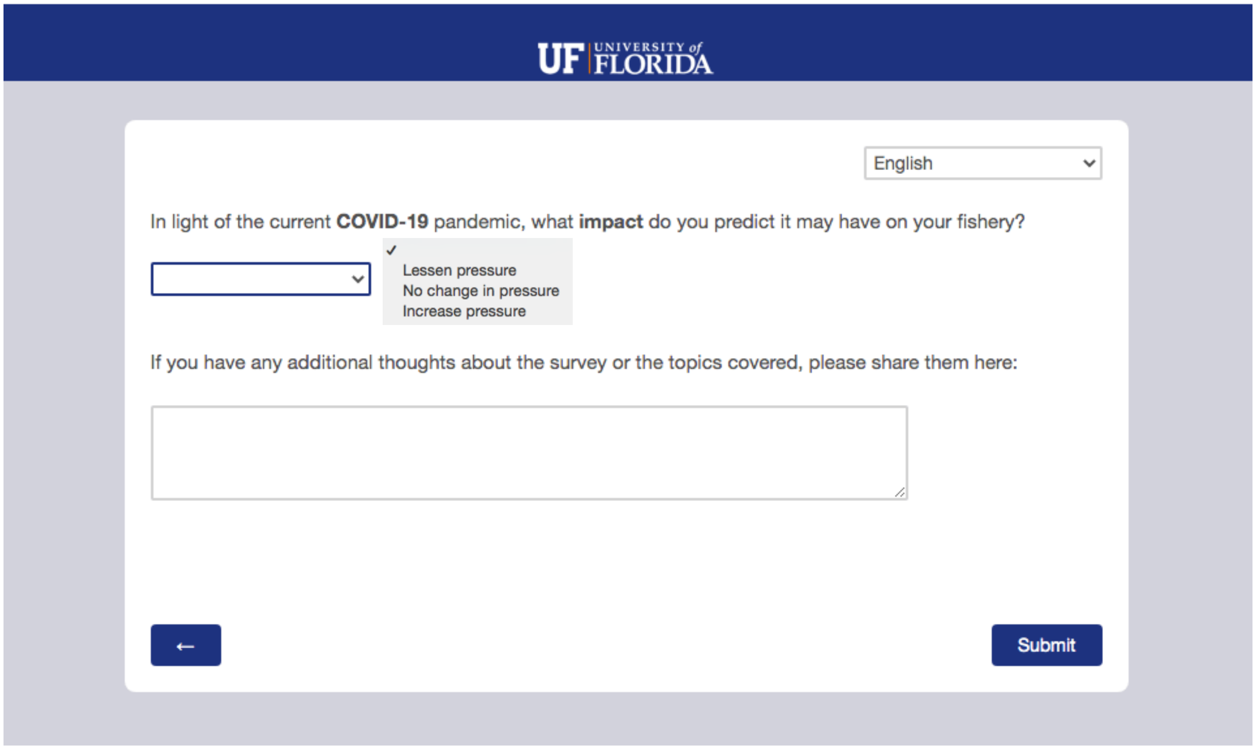
UF UNIVERSITY of FLORIDA

English

In light of the current **COVID-19** pandemic, what **impact** do you predict it may have on your fishery?

✓
Lessen pressure
No change in pressure
Increase pressure

If you have any additional thoughts about the survey or the topics covered, please share them here:

←

Submit
